# Health care registers can be instrumental for endpoint capture in clinical diabetes trials: example of microvascular complications in Swedish patients with type 2 diabetes

**DOI:** 10.1177/14791641231179878

**Published:** 2023-06-15

**Authors:** Martin H Lundqvist, Vagia Patsoukaki, Stefan Jansson, Henrietta Norman, Elisabet Granstam, Maria K Svensson, Johan Sundström, Björn Eliasson, Jan W Eriksson

**Affiliations:** 1 Department of Medical Sciences, 8097 Uppsala University, Uppsala, Sweden; 2 Faculty of Medicine and Health, University Health Care Research Center, 6233 Örebro University, Örebro, Sweden; 3 Department of Public Health and Caring Sciences, 8097 Uppsala University, Uppsala, Sweden; 4 Center for Clinical Research Sörmland, 8097 Uppsala University, Eskilstuna, Sweden; 5 Center for Clinical Research, 8097 Uppsala University/Region Västmanland, Västerås, Sweden; 6 The George Institute for Global Health, University of New South Wales, Sydney, Australia; 7 Department of Medicine, Sahlgrenska University Hospital, Gothenburg, Sweden; 8 National Diabetes Register, Centre of Registries in Region Western Sweden, Gothenburg, Sweden

**Keywords:** Type 2 diabetes, microvascular complications, diabetes register, validation, register-based randomized clinical trial, endpoint

## Abstract

**Aims:**

SMARTEST is a register-based randomized clinical trial (RRCT) that compares dapagliflozin to metformin in early-stage type 2 diabetes. The primary outcome includes progression of microvascular complications based on data from the Swedish National Diabetes Register (NDR). In this sub-study, the aim was to validate microvascular complication variables in the NDR against electronic health records (EHRs).

**Methods:**

Data were extracted from EHRs of 276 SMARTEST participants with a median observation period of 3 years in the Uppsala, Örebro and Sörmland counties and compared with NDR data. Agreement was determined for all corresponding data entries as well as for progression of microvascular complications after randomization.

**Results:**

The agreement for all corresponding data entries was 98.9% (Intraclass Correlation Coefficient 0.999) for creatinine and eGFR, 95.1% for albuminuria, 91.6% for foot-at-risk and 98.2% for retinopathy status (Kappa 0.67-0.91). The agreement for progression of microvascular complications was 98.0% for CKD stage, 98.9% for albuminuria grade, 96.3% for foot-at-risk grade and 99.6% for retinopathy grade progression (Gwet’s AC_1_ 0.96-1.00).

**Conclusion:**

Microvascular complication variables in the NDR show good agreement with EHR data. The use of a well-established national health care registry, exemplified by the NDR, for endpoint collection in RRCTs such as SMARTEST is supported by this study.

## Key messages

• Data on microvascular in the Swedish National Diabetes Register (NDR) display good agreement with data from electronic health records
• The ascertainment of the NDR was particularly good for retinopathy and nephropathy
• Within nephropathy, there was 98.9% concordance for eGFR and 95.1% for albuminuria
• For diabetic foot problems, the concordance was lower but acceptable
• The NDR is suitable for use in register-based clinical trials

## Introduction

Chronic hyperglycemia in type 2 diabetes (T2D) leads to micro- and macrovascular complications that greatly impact the longevity, morbidity and quality of life for people with diabetes.^1–3^ SGLT2i have consistently been shown to effectively prevent the development and progression of several diabetes complications when used as add-on treatment for T2D in high-risk individuals.^4–8^ In the ongoing SMARTEST study, conducted in Sweden, the SGLT2i dapagliflozin is compared to metformin for treatment of early-stage T2D.^9^ The primary composite endpoint includes development or progression of micro- and macrovascular diabetes complications and are based on information obtained from national registers. The RRCT study design thus adopted is a cost-effective alternative to the traditional RCT, and has been used to an increasing extent in several medical fields.^10^ While observational register studies are common within the field of diabetes,^11,12^ the SMARTEST study is to our knowledge the first RRCT to be conducted within this field.

In the SMARTEST study, the Swedish National Diabetes Register (NDR) is the source of several endpoints, including development or progression of microvascular complications. This national quality register includes approximately 90% of all patients with diabetes in Sweden and while it was originally launched to monitor quality in Swedish diabetes care, it has also been extensively used in research.^13^ In similarity with many other registers, the validity of data in the NDR has not been ascertained, which could have consequences for the interpretability of the SMARTEST study.

In this study, the aim was to evaluate the concept of the RRCT by validating clinical data in the NDR. This was done by investigating the agreement between data captured in the NDR and electronic health records (EHRs), specifically regarding the development or progression of microvascular complications, for a subset of the SMARTEST study population.

## Methods

SMARTEST is a nationwide study with more than 30 study sites. The participants are randomized 1:1 to dapagliflozin or metformin and are then followed in regular care. Periodical withdrawals from national registers, including NDR, are made for endpoint collections. Adults with T2D according to WHO criteria with less than 4 years duration and BMI 18.5–45 kg/m^2^ that are either drug naive or are receiving monotherapy for their diabetes are included. Exclusion criteria include more than 4 weeks treatment with insulin, GLP1-RA, SGLT2i or any combination of glucose-lowering medications, HbA1c > 70 mmol/mol on monotherapy and >80 mmol/mol for drug naïve, established cardiovascular disease, renal failure (eGFR<60 mL/min/1.73 m^2^), pregnancy or breastfeeding.

This sub study to SMARTEST was conducted in the Uppsala, Örebro and Sörmland counties. For participants included in the SMARTEST study and residing within the respective county, data was manually extracted from EHRs between 7 April 2021 and 30 March 2022 and compared to data extracted from the NDR on 8 June 2022. Participants were generally included in the order of inclusion in the SMARTEST study in each county, with some exceptions due to work distribution among the study personnel. The participants had consented to data collection from EHRs when signing the written informed consent to the SMARTEST study, which has received approval from the Swedish Ethical Review Authority (DNR 2019-01747).

### Data extraction from EHR

Data regarding diabetes complications from the date of diagnosis for each participant were documented in a structured eCRF using RedCap^14^ hosted by Uppsala University. The data extraction was performed by qualified health personnel with no scientific interest in the SMARTEST study at the Uppsala University Hospital, the Clinical Research Center at Örebro and at Mariefred Primary Health Care Center in Sörmland. Documented variables included creatinine, UACR, foot examinations (monofilament test, tuning fork test, pulse palpation), fundus examination (retinopathy status and stage) and corresponding dates for these examinations and blood tests. Each participant was reviewed from the date of his/her diabetes diagnosis and onwards, except for fundus examinations, that were reviewed from 18 months before randomization and onwards. The participant’s study-ID number was used for identification and no other identifying information were entered in the eCRF.

### Data extraction from the NDR

Health care units that manage patients with T2D are expected to report individual clinical data for these patients at least annually to the NDR. Within the framework of the SMARTEST study, withdrawals from the NDR are made every 3 months and stored in a central study database, operated by the Uppsala Clinical Research Center. In this sub study, NDR data withdrawn on 8 June 2022 were obtained from this database for the included participants. These data included creatinine, eGFR (MDRD^15^ and CKD-EPI^16^ equations), albuminuria grade, numerical value of UACR, foot-at-risk grade, status and grade of retinopathy. In the NDR, creatinine is reported as a continuous variable from which eGFR is calculated based on age and gender. Albuminuria is graded in 0–3 (0 = no albuminuria, 1 = normalized albuminuria (after treatment), 2 = microalbuminuria, 3 = macroalbuminuria) and since 2019 also optionally as numerical value in mg/mmol. Foot-at-risk grade is reported as 1-4 (1 = healthy foot 2 = signs of neuropathy/angiopathy, 3 = previous history of severe foot complications and 4 = ongoing severe foot complications) based on patient history and clinical examination. Retinopathy is reported as status (no = 0, yes = 1) and grade 1-4 (1 = mild, 2 = moderate, 3 = severe and 4 = proliferative retinopathy). For foot-at-risk grade and retinopathy, the date on which the examinations were made is documented, usually specified as the first day of the month. For all register entries, the date of entry is documented.

### Data management

**Figure 1.**
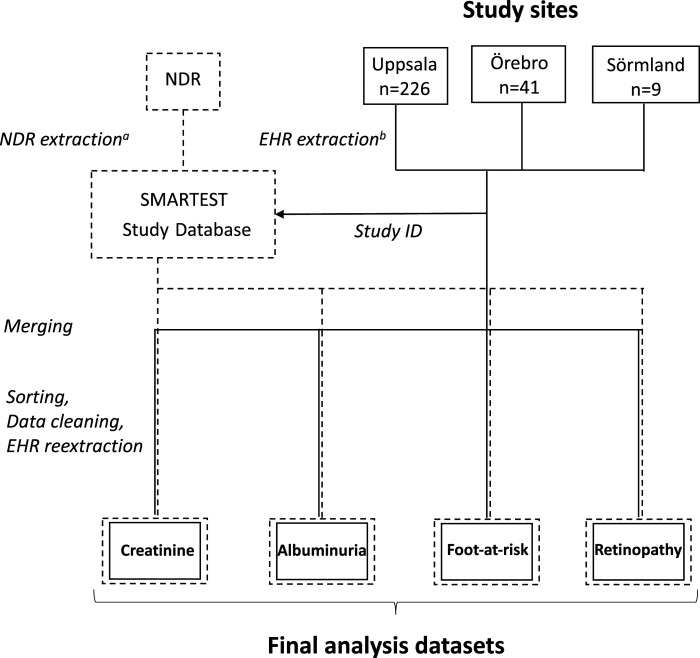
Data flow and management Solid boxes and arrows represent location and flow of EHR (electronic health records) data. Dashed boxes and arrows represent location and flow of NDR (the Swedish National Diabetes Register) data. ^a^Extracted on 8 June 2022^ b^Extracted manually between 7 April 2021 and 31 March 2022.

A scheme of data flow and management is provided in Figure 1. Data from EHR and the NDR were split into four categories: creatinine/CKD stage, UACR, foot-at-risk and retinopathy. For each of these categories, data from EHRs and the NDR were merged and sorted by study-ID and date. Data from the NDR were then linked to and paired with data from EHRs based on date of examinations and/or register entry and for continuous variables also the numerical value of the variable. Only NDR data that had been registered or were referring to source data within the observational period were included. Once data had been merged, sorted and paired, the four datasets were inspected and in cases of very discrepant information in EHRs versus the NDR, a targeted reextraction of EHRs was undertaken, after which data was confirmed, corrected, removed or added. Importantly, all authors remained blinded to information on randomized study treatment since this was not included in the analysis datasets.

### Classification of data on microvascular complications

Creatinine was handled as a continuous variable for the purpose of validation and was not processed further. For albuminuria, categorization of EHR data was made based on the following algorithm: normal was defined as UACR<3 mg/mmol; microalbuminuria was defined as an incident UACR of 3–30 mg/mmol; macroalbuminuria as an incident UACR >30 mg/mmol. Due to lack of impact for the SMARTEST study, no differentiation was made between no albuminuria and normalized albuminuria (after treatment) which were merged into the same category. For NDR data, the reported albuminuria grade or the transformed albuminuria grade, based on the numerical UACR and by the same algorithm as for EHR data, was utilized. In cases where both an albuminuria grade and a numerical value had been entered; supremacy was considered for the numerical value.

For foot-at-risk, guidelines from the NDR and the Swedish Association of Local Authorities and Regions^17^ were adopted. Thus, grade 1 was defined as the absence of abnormalities in monofilament test, tuning fork test or pulses and no manifest diabetes-related foot lesions, e.g. ulcers. Grade 2 was defined as abnormal findings in at least one of monofilament test, tuning fork test or pulses. Grade 3 was defined as signs of neuropathy or angiopathy according to grade 2 plus history of ulcers, amputation, deformities, or skin fissures. Grade 4 was defined as ongoing ulcers, osteopathy or neuropathic pain. For NDR data, the foot-at-risk grade was utilized without further processing.

Retinopathy status (yes/no) was based on the fundus examination in EHR and on the retinopathy status and grade variables in the NDR. The retinopathy grades (mild, moderate, severe and proliferative) provided in the fundus examination of EHR and the NDR were utilized without further processing.

### Definition of microvascular events: Development or progression of microvascular complications

Preliminary definitions of microvascular events in the SMARTEST study were adopted.

Thus, progression of CKD stage was defined as a decline in eGFR, calculated with the 2009 CKD-EPI formula,^16^ from above to below 60 mL/min/m^2^ (CKD stage 3) observed >12 months after randomization that was either repeatedly observed on at least one consecutive non-identical observation or the last available observation. The rationale behind the 12-month limit is the well-described temporary drop in eGFR that occurs immediately after treatment initiation with SGLT2-inhibitors and remains up to 12 months compared with placebo.^8,18,19^

Development or progression of albuminuria, foot complications and retinopathy were defined as the progression to a higher stage (i.e. from no albuminuria to micro-/macroalbuminuria or from micro-to macroalbuminuria) from the highest of the inclusion visit assessment and the last NDR entry or EHR data, respectively, within 18 months that was either repeatedly observed on at least one consecutive and non-identical observation OR the last available observation OR, for albuminuria, normalized after initiation of RAAS-blockade therapy. Observations were only counted as events if > 30 (>90 for retinopathy) days from randomization to date of examinations (foot-at-risk, retinopathy) or register entry (albuminuria). For foot-at-risk and retinopathy, the date of examinations was converted to the 15^th^ of the month, if registered as the 1^st^ of that month. The analysis of microvascular events was restricted to participants with a follow-up time of more than the above specified time limits, i.e. to participants at risk to have the events.

### Statistical analysis

General aspects of data quality were presented descriptively. This included source specific data distribution and overlap, completeness (percentage of EHR data transferred to the NDR), uniqueness (percentage of NDR data that could be linked to EHR data), duplicate and extraneous NDR data, reporting rate (number of unique NDR entries per patient year) and timeliness between corresponding NDR (date of entry or examination utilized as applicable) and EHR entries.

Validation was carried out for corresponding entries in the NDR and EHRs as well as for microvascular events based on NDR versus EHR data. EHR data were considered the golden standard. For the continuous variable creatinine, the exact percentual agreement in the NDR versus EHRs and the single measure type ICC, using a two-way mixed model with an absolute agreement definition^20^ was determined. For categorical variables, the exact agreement and Cohen’s kappa^21^ (κ, for dichotomous variables) or Cohen’s weighted kappa with linear weights^22^ (κ_w_, for polytomous ordered variables) were determined. The following cutoffs for interpretation of kappa values have been proposed: 0.01-0.20 = poor, 0.21-0.40 = fair, 0.41-0.60 = moderate, 0.61-0.80 = good, 0.81-1 = very good.^23^ In cases with low prevalence of some categories, Gwet’s AC_1_,^24^ that provides more robust estimates in this setting,^25^ was used instead. In addition, for dichotomous variables, sensitivity, specificity, positive predictive value (PPV) and negative predictive value (NPV) were determined. Excel for Mac version 16.66.1 (Microsoft corporation, Redmond, WA) was used for data handling. ICC was calculated in SPSS v28.0 (IBM corp., Armonk, NY). Kappa and AC_1_ estimates were calculated in R Statistical Software v 4.2.2 (R Core Team, 2021) using the irrCAC R Package v1.0 (Gwet 2019). The AC_1_ estimates were assessed in terms of the Altman benchmarking scale^23^ presented above, using the function altman.bf (). Descriptive plots were constructed in Graph Pad Prism version 9.1.0 (GraphPad Software, San Diego, Ca).

## Results

### Participant characteristics

This subset of SMARTEST study participants included 276 individuals with a combined observational period of 776 patient-years (median 3 years per participant), of which 271 patient-years were after randomization in the SMARTEST study (median 13 months per participant). They were included in study centers within the defined geographical area, from 5 September 2019 to 7 February 2022. The majority were men (180; 65.2%). At inclusion in the SMARTEST study, they were generally overweight/obese (BMI 30.8 ± 5.1 kg/m^2^), had early-stage type 2 diabetes (duration 21 ± 15 months) with good glycemic control (HbA1c 45.7 ± 8.0 mmol/mol) and low prevalence of diabetes complications (albuminuria 9.8%, neuropathy/angiopathy 3.6%, retinopathy 9.4%). Detailed information on clinical characteristics at inclusion in the SMARTEST study is provided in Table 1.

**Table 1.** Participant characteristics (*n* = 276) at inclusion in a regional subset of the SMARTEST study. Data presented as a *n* (%) or mean ± SD. Treatment refers to before inclusion.

Variable	*n* = 276
Gender, male/female (%)	180/96 (65.2/34.8)
Age, years	61 ± 10
T2D duration, months	21 ± 15
BMI, kg/m^2^	30.8 ± 5.1
Systolic blood pressure, mmHg	139 ± 16
Diastolic blood pressure, mmHg	84 ± 10
HbA1c, mmol/mol	45.7 ± 8.0
Creatinine μmol/l	74.5 ± 13.6
eGFR, ml/min/1.73 m^2^	88.8 ± 14.1
Total cholesterol, mmol/l	4.8 ± 1.1
LDL, mmol/l	2.98 ± 0.98
HDL, mmol/l	1.29 ± 0.34
Triglycerides, mmol/l	2.04 ± 1.26
Nephropathy, none/micro-/macroalbuminuria (%)	249/26/1 (90.2/9.4/0.4)
Neuropathy/Angiopathy, no/yes (%)	266/10 (96.4/3.6)
Retinopathy^a^, no/yes (%)	229/26 (83.0/9.4)
Retinopathy grade, mild/mod/sev/prol (%)	22/3/1/0 (8.0/1.1/0.4/0)
Metformin, *n* (%)	236 (85.5)
Drug naïve, *n* (%)	40 (14.5)
Antihypertensive treatment, *n* (%)	185 (67.0)
ACEI, *n* (%)	53 (19.2)
ARB, *n* (%)	93 (33.7)
Lipid-lowering agent, *n* (%)	117 (42.4)

^a^Baseline assessment missing for 21 subjects. mod = moderate, sev = severe, prol = proliferative, ACEI = Angiotensin-Converting-Enzyme Inhibitors, ARB = Angiotensin II Receptor Blockers.

### General data characteristics

Table 2 displays the distribution of data in the NDR and EHRs. NDR completeness (percentage of EHR entries transferred to the NDR) was 54.7% for creatinine, 55.3% for albuminuria, 66.6% for foot-at-risk and 86.7% for retinopathy status. NDR uniqueness (percentage of NDR entries that could be linked uniquely to EHR entries) was 66.3% for creatinine, 72.5% for albuminuria, 66.8% for foot-at-risk and 44.8% for retinopathy status, the remaining NDR data consisting predominantly of duplicate NDR entries (Table 2). Unreported, missing data from EHR did not differ from EHR data that had been reported to the NDR, with the exception of foot-at-risk, where the proportion of neuropathy/angiopathy was significantly higher among reported data (Supplement S1). Out of the valid NDR entries as retinopathy = yes, there was an associated retinopathy stage in 43.3% (13/30), but this reporting rate increased during the observational period, exceeding 80% by 2022 (Supplement, S2). The reporting rate of unique NDR entries per patient year was 1.09 for creatinine, 0.71 for albuminuria, 0.86 for foot-at-risk and 0.36 for retinopathy. The median number of total unique entries per participant was 3 for creatinine, 2 for albuminuria grade, 2 for foot-at-risk grade and 1 for retinopathy. The number of participants that had at least one unique NDR entry was 264 (95.7%) for creatinine, 237 (85.9%) for albuminuria, 216 (78.3%) for foot-at-risk and 237 (85.9%) for retinopathy. Counting only unique entries after randomization, the equivalent numbers were 200 (72.5%) for creatinine, 151 (54.7%) for albuminuria, 161 (58.3%) for foot-at-risk and 116 for retinopathy (42.0%) (Supplement S3 and S4). The median time between EHR and NDR entry was 7 days for creatinine, 6 days for albuminuria and 0 days for foot-at-risk and retinopathy.

**Table 2.** Data distribution for entries in the NDR and EHRs.

	Creatinine	Albuminuria	Foot-at-risk	Retinopathy
EHR, total	1548	996	1007	323
EHR + NDR^a^	846	551	671	280
NDR, total	1276	760	1004	625
NDR, duplicate entries^b^	315	145	277	330
NDR, extraneous entries^c^	115	64	56	15

^a^NDR and EHR data that could be linked uniquely,

^b^repeated entries without new information appearing in EHR,

^c^NDR entries that could not be linked to available EHR data.

### Agreement of corresponding NDR and EHR entries

The agreement was 98.9% (ICC 0.999, 95% CI 0.999-0.999, *p* < 0.001) for a total of 846 corresponding creatinine entries, 95.1% (κ_w_ 0.80, 95% CI 0.72-0.87, *p* < 0.001) for the 551 corresponding albuminuria grade entries, 91.6% (κ 0.67, 95% CI 0.59-0.76, *p* < 0.001) for the 606 corresponding foot-at-risk grade entries and 98.2% (κ 0.91, 95% CI 0.84-0.99, *p* < 0.001) for the 280 corresponding retinopathy status entries. Table 3 displays crosstabulations of entries in the NDR versus EHRs with associated sensitivity, specificity, PPV and NPV, where applicable. For the 13 corresponding data on retinopathy grade (11 mild, 2 moderate), the agreement was 100% (κ 1.00, 95% CI 1.00-1.00, *p* < 0.001).

**Table 3.** Categorical classification of microvascular complications in corresponding NDR and EHR entries.

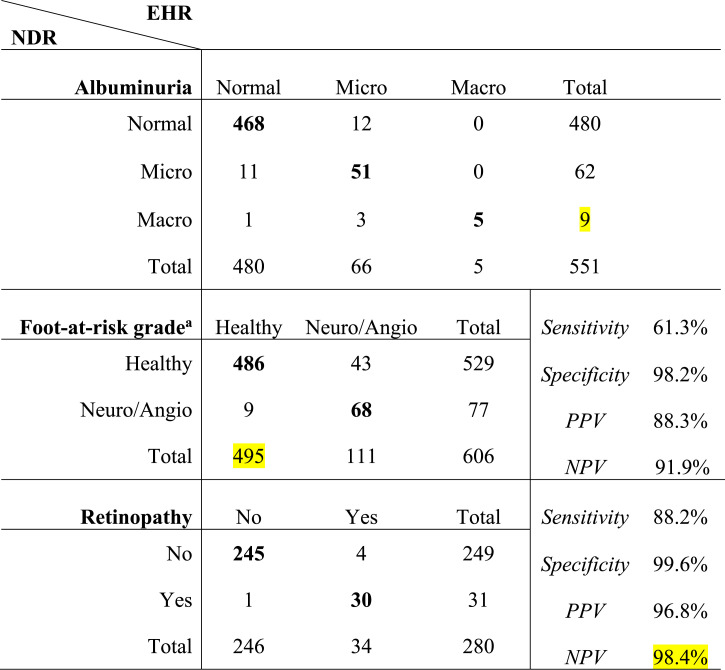

^a^EHR datapoints with an assessment of foot-at-risk grade but with no information on clinical examinations were not included in the analysis. Neuro = neuropathy, Angio = angiopathy, PPV = positive predictive value, NPV = Negative predictive valueAgreement of microvascular events fulfilment according to the NDR versus EHRs.

The agreement between the two sources was assessed for events of development or progression of microvascular complications. It was 98.0% (AC_1_ 0.98, 95% CI 0.95-1.00, *p* < 0.001) for CKD stage, 98.9% (AC_1_ 0.99, 95% CI 0.97-1.00, *p* < 0.001) for albuminuria, 96.3% (AC_1_ 0.96, 95% CI 0.93-0.99, *p* < 0.001) for foot-at-risk and 99.6% (AC_1_ 1.00, 95% CI 0.99-1.00, *p* < 0.001) for retinopathy progression. All these agreement estimates were classified as very good, according to Altman’s benchmarking scale.^23^Table 4 displays crosstabulations of microvascular events according to the NDR versus EHRs with associated sensitivity, specificity, PPV and NPV.

**Table 4.** Microvascular events based on data in the NDR versus EHRs.

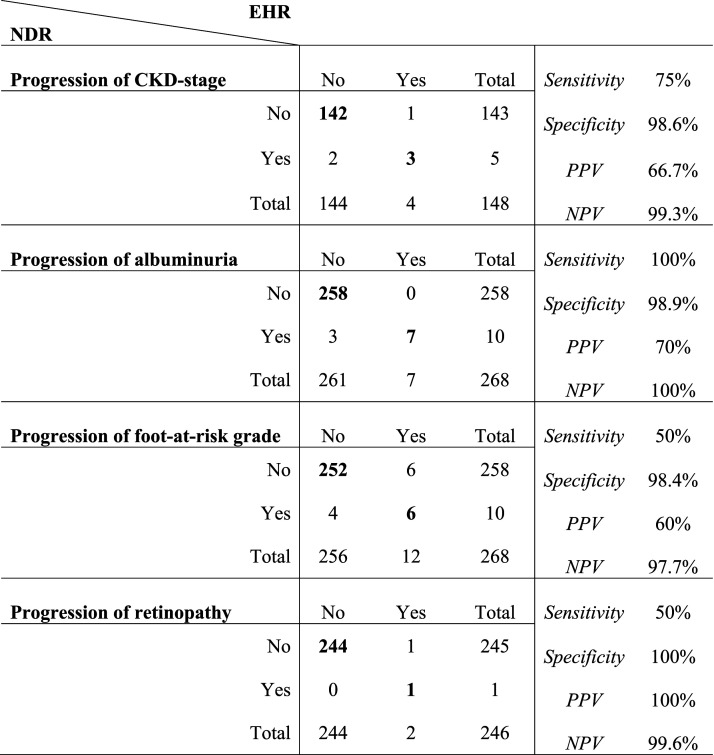

PPV = positive predictive value, NPV = negative predictive value.

## Discussion

In this study, we have validated variables pertaining to microvascular complications in the NDR against source data in EHRs. We found that the agreement between corresponding NDR and EHR entries was good concerning albuminuria and foot-at-risk grade and very good concerning creatinine and retinopathy status. Since this was a sub study to the SMARTEST study, we also evaluated the agreement between NDR and EHR data regarding fulfilment of microvascular events as preliminarily defined in the SMARTEST study. In this context, the agreement was very good for all events: progression of CKD-stage, albuminuria grade, foot-at-risk grade and retinopathy stage. The completeness of NDR data was adequate, entries based on novel information in EHRs occurring at average more than annually for creatinine, slightly less than annually for albuminuria and foot-at-risk and more than once every three years for retinopathy; all in keeping with Swedish national guidelines.^26^ In general, the assessable date of NDR data was timely with respect to appearance in EHRs; the delay was equal to or less than a week for all variables at median. Although clinical data in the NDR have been previously validated, this is the first publication of the validity of microvascular complication variables in the NDR. The findings reinforce the use of the NDR for observational studies and support its use as source data in RRCTs such as the SMARTEST study.^9^

### General data characteristics

The missingness of data in the NDR with respect to EHRs was overall quite substantial; around 45% for creatinine and albuminuria, 33% for foot-at-risk grade, while only 13% for retinopathy status. Corresponding retinopathy grades were only available for 43% of all retinopathy entries but increased during the observational period, probably reflecting increasing awareness of the variable since its recent introduction in 2018. Creatinine and UACR may be obtained for reasons other than routine surveillance of diabetes complications by other health care units that do not report to the NDR. This is far less likely regarding foot examinations and highly implausible regarding fundus examinations. Moreover, diagnosis of albuminuria is often, and should be, a congregated assessment based on several repeated measurements. Previous measurements that have not coincided with the reporting of the albuminuria grade may therefore have been factored into the assessment notwithstanding the absence of a temporal association. Human errors in the documentation or transfer of EHR data or patient objection to data transfer may also explain some data loss to the NDR. Importantly, routines regarding frequency, completeness and method of reporting to the NDR differ among health care providers. For example, around one-third of all health care units still employs manual reporting of NDR data and automated data extraction, when implemented, is usually limited to the last available data for each variable. Obviously, a less selective automated extraction process, standardized and employed by all health care providers would overcome many potential sources of data loss. However, such a procedure may have ethical and legal issues relating to personal integrity.

Notwithstanding possible causes of the observed missingness, there were no substantial numerical or proportional differences between missing and reported data, suggesting that neither the current results, nor the reliability of future results from the SMARTEST study appear to be at risk of marked influence from systematic, non-random loss of real-world data. Taken together, the resulting reporting rate for each investigated variable in the NDR seems adequate, both for the primary purpose of monitoring the national quality of diabetes care as well as for the applicable use as source data in observational and interventional clinical studies.

### Agreement of microvascular complications and events in the NDR versus EHR

Not surprisingly, the agreement between the NDR and EHRs was best for the more objective variables creatinine and retinopathy. For these variables, the minute discrepancies between the two sources were probably attributable to the human factor in EHR documentation or transfer to the NDR. Albuminuria is predominantly reported to the NDR as a grade based on the UACR value at one or several occasions. Thus, the quality of this variable is ultimately dependent on the clinicians’ judgement. Ignorance about the cut-offs between albuminuria grades may be one source of error. Clinicians may also see variant need for repeated measures of UACR before making the diagnosis of albuminuria. According to guidelines, albuminuria should be diagnosed if indicated on 2 out of 3 measurements within 3–6 months.^27^ Most likely, departures from these guidelines are made on a regular basis in clinical practice. Nevertheless, more than half of all NDR entries as albuminuria of any stage and almost 80% of repeated NDR albuminuria entries with available preceding EHR information were based on 2/3 UACR measurements in the albuminuria range. The built-in requirement of confirmatory NDR entries in the preliminary definition of albuminuria events thus ensures that these events are well-grounded and the demonstrated excellent agreement between albuminuria progression based on NDR versus EHR data further corroborates the operability of this preliminary definition.

Regarding foot-at-risk grade, national guidelines from The Swedish Association of Local Authorities and Regions^17^ state that any pathologic finding with regards to tuning fork test, monofilament test or pulse palpation should prompt a foot-at-risk grade 2. In clinical practice however, clinicians may value findings differently and make an overall assessment of the foot-at-risk grade. In particular, negligence to report isolated abnormalities in tuning fork test were the cause of inconsistency between the sources in almost 80% of cases of false negative foot-at-risk grades. Abnormal findings on the monofilament test were less frequently disregarded. While the tuning fork test may be more sensitive than the monofilament test in detecting neuropathy,^28,29^ results from the monofilament test have been indicated to better predict the risk to develop diabetic foot ulcers.^30,31^ From this perspective, the supreme emphasis on the monofilament test in the clinical evaluation of the foot-at-risk grade appears well-grounded. Furthermore, when comparing documented risk foot grade in corresponding NDR and EHR entries, the agreement between these sources was close to perfect (Supplement S5). Thus, it is fair to state that the NDR appears to accurately reflect the clinician’s overall assessment of the foot-at-risk grade, while this assessment may not be in full agreement with the documented findings on clinical examination.

### Comparison with other health-care registers

The SWEDEHEART^32^ register has been employed in numerous published and ongoing register-based randomized controlled trials.^33–35^ According to annual internal quality control, SWEDEHEART variable agreement with EHR data has been reported to be in the range 92.4–97.4%,^32,36^ which is in line with the agreement of NDR variables as presented in this study. The agreement is also similar to the Swedish Fracture Register,^37^ which is being employed in several ongoing RRCTs.^38,39^

### Strengths and limitations

This is the first attempt to validate microvascular complication variables of the NDR. We have studied a large population with a long combined observational period. The extraction of EHR data has been systematic and has occurred independently of NDR data by independent health care staff with no self-interest in the outcome of the study.

There are however some important limitations that need to be addressed. First, many participants did not have unique NDR entries after randomization and accordingly, the number of events for validation were few. Following inclusion in the SMARTEST study, the study participant’s regular care provider is notified regarding study involvement and is actively encouraged to report to the NDR. Thus, there is no considerable risk that this sparsity of NDR entries after randomization would result in an overestimation of the agreement between corresponding data from the two sources. Regarding agreement of event fulfilment, we recognize the need for further validation at a later stage of the SMARTEST study. However, it seems likely that discrepancies between the two sources would be expected to diminish rather than enhance with prolonged follow-up time and accumulation of data. Second, some EHR data may have been overlooked due to complicated structure of the EHRs. There are also some private care providers whose EHRs are difficult to access for external healthcare personnel. Third, the cohort in this study was limited to participants residing in three out of twenty-one Swedish health care regions, amounting to approximately one third of the SMARTEST study population that had been recruited by the time the current EHR extraction was terminated. Although this selected cohort was highly representative of the nationwide SMARTEST population in terms of clinical characteristics,^40^ neither the participants nor the primary care centers these participants attended are likely to fully reflect real-world diabetes care in Sweden. However, in the geographical area we studied there is a mixture of rural and urban residency, socioeconomic strata and private and public primary care centers, and this is similar to the whole country. Nevertheless, confirmatory validation in a less selected cohort of patients with type 2 diabetes and corresponding regular care providers may be warranted. Fourth, our analysis was limited to microvascular diabetes complications. The primary composite endpoint in the SMARTEST study also includes macrovascular complications and all-cause death, which are based on information from the National Patient Register and the Swedish Population Register, respectively. While validation of the implementation of these registers in the SMARTEST study is planned, it was not done at the time of the current analysis due to the scarcity of such events.

Regardless of the limitations it is likely that the validity of NDR’s microvascular complication data would be similar across Swedish primary care for type 2 diabetes. Importantly, a main aim of this study was to address whether the NDR would provide appropriate data to collect outcome events of microvascular complications in the specific context of a register-based randomized controlled trial. We find that the NDR is a robust and valid data source for this purpose. Nonetheless, further research is warranted, for example to address the validity of register data of long-term trajectories of diabetes complications. We hope that this study may serve as an example for validation of register data that may be extended to other local and national health registers that are employed both for monitoring of health care quality and for scientific purposes.

## Conclusions

In summary, this is the first published study validating data on microvascular complications from the Swedish National Diabetes Register (NDR). We found that they largely display very good agreement with source data from EHRs. This is comparable to other Swedish registers used in RRCTs. RRCTs offer a cost-effective way to evaluate clinical interventions in a real world setting, and they are being conducted to an increasing extent in several disease areas.^10^ To our knowledge, the SMARTEST study is the first RRCT to be conducted within the field of diabetes. This validation study reinforces the use of the NDR in observational studies and also provides robust support for the NDR as source data in the ongoing RRCT SMARTEST as well as other future RRCTs.

## Supplemental Material

Supplemental Material - Health care registers can be instrumental for endpoint capture in clinical diabetes trials: example of microvascular complications in Swedish patients with type 2 diabetes Click here for additional data file. Supplemental Material for Health care registers can be instrumental for endpoint capture in clinical diabetes trials: example of microvascular complications in Swedish patients with type 2 diabetes by Martin H Lundqvist, Vagia Patsoukaki, Stefan Jansson, Henrietta Norman, Elisabet Granstam, Maria K Svensson, Johan Sundström, Björn Eliasson and Jan W Eriksson in Diabetes & Vascular Disease Research

## Appendix

### Abbreviations

CKD: Chronic Kidney Disease
eCRF: Electronic Case Report Form
eGFR: estimated Glomerular Filtration Rate
EHR: Electronic Health Record
GLP-1 RA: Glucagon-Like Peptide 1 Receptor Agonist
Gwet’s AC_1_: Gwet’s First-order Agreement Coefficient
ICC: Intraclass Correlation Coefficient
κ: Cohen’s Kappa
κ_w_: Cohen’s Kappa with linear weights
NDR: Swedish National Diabetes Register
NPV: Negative Predictive Value
PPV: Positive Predictive Value
RAAS: Renin-Angiotensin-Aldosterone System
RCT: Randomized Controlled Trial
RedCap: Research Electronic Data Captures
RRCT: Register-based Randomized Clinical Trial
SGLT2i: Sodium-glucose transporter 2 inhibitors
SMARTEST: SGLT2 inhibitor or metformin as standard treatment of early stage type 2 diabetes
SWEDEHEART: Swedish Web-system for Enhancement and Development of Evidence-based care in Heart disease Evaluated According to Recommended Therapies
T2D: type 2 diabetes
UACR: Urinary Albumin-Creatinine Ratio
