# Supplementary material for: Health care registers can be instrumental for endpoint capture in clinical diabetes trials: example of microvascular complications in Swedish patients with type 2 diabetes
Source: Diab Vasc Dis Res. 2023 Jun 15;20(3):14791641231179878. doi: 10.1177/14791641231179878 (PMC10286550; doi:10.1177/14791641231179878)
Supplement: Supplemental Material - Health care registers can be instrumental for endpoint capture in clinical diabetes trials: example of microvascular complications in Swedish patients with type 2 diabetes [file sj-pdf-1-dvr-10.1177_14791641231179878.pdf]

S1 Description of missing data vs reported data from EHR to NDR

Variable		EHR only	EHR + NDR	*p*
Creatinine, µmol/l		76±16 (n=702)	75±14 (n=846)	0.124[a]
Albuminuria, n (%)	Normal	380 (85.6)	481 (87.1)	
	Micro	60 (13.5)	66 (12.0)	
	Macro	4 (0.9)	5 (0.9)	0.758[b]
Foot-at-risk, n (%)	Healthy	299 (89.0)	547 (81.5)	
	Neuro/angio	37 (11.0)	124 (18.5)	0.003[b]
Retinopathy, n (%)	None	37 (86.0)	246 (87.9)	
	Mild	6 (14.0)	30 (10.7)	
	Moderate	0	2 (0.7)	
	Severe	0	2 (0.7)	
	Proliferative	0	0	0.776 [b]

[a]t-test with equal variances not assumed, [b]Fisher's exact test

S2 Missingness of associated retinopathy grade in NDR entries with reported presence of retinopathy

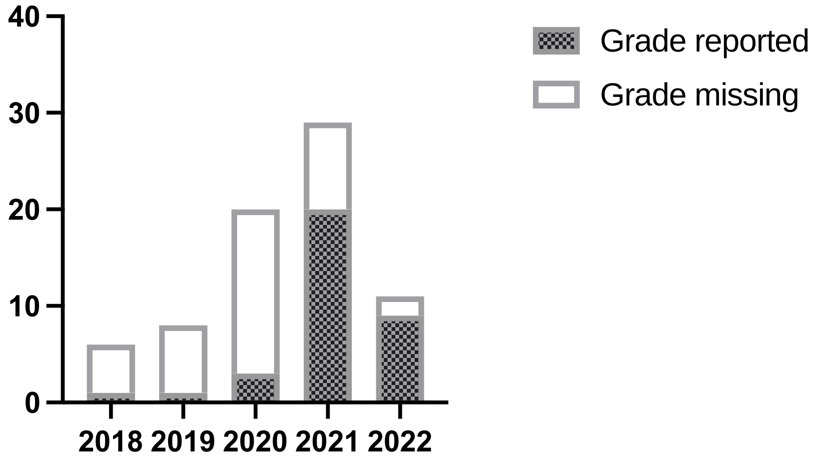

S3 Number of unique NDR entries during the whole observational period per participant

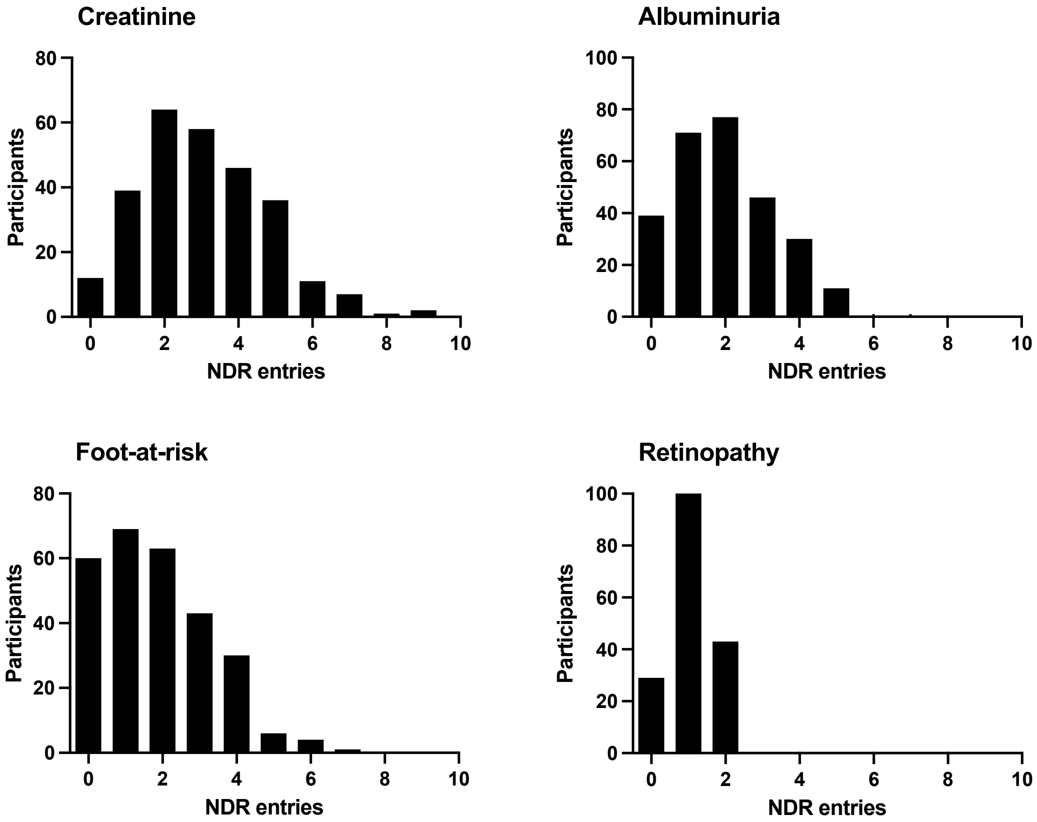

S4 Number of unique NDR entries after randomization in the SMARTEST study per participant

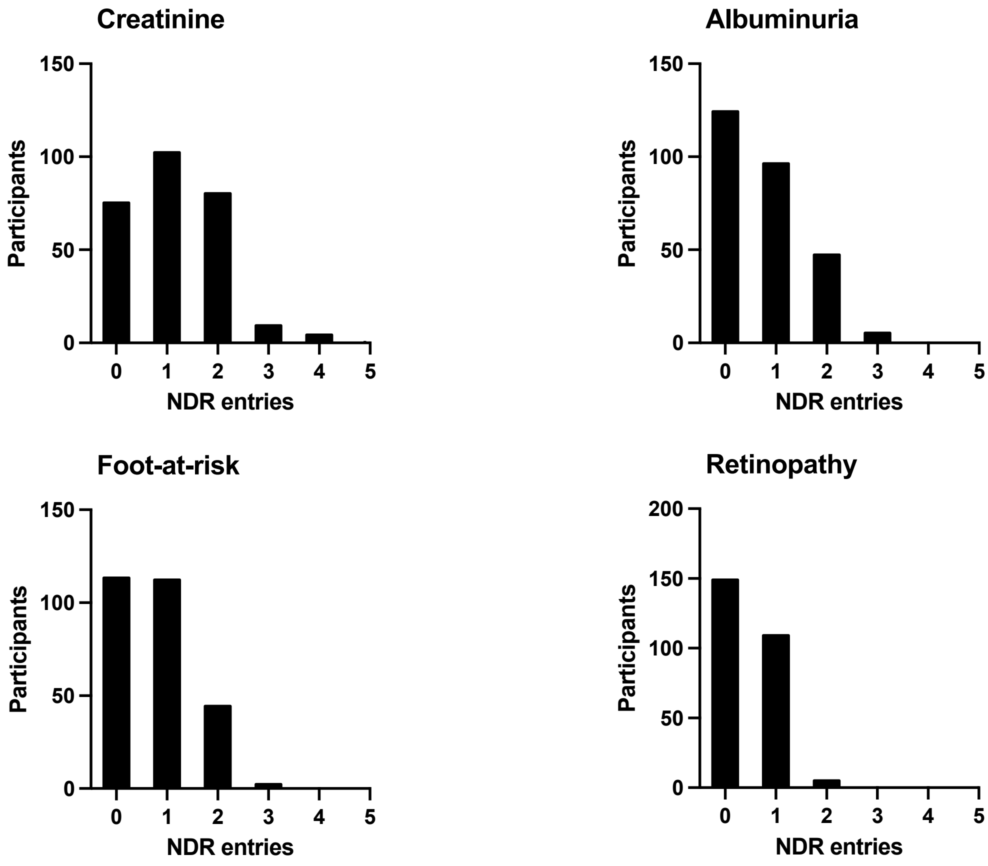

S5 Foot-at-risk stage assessment in NDR vs EHR

NDR \ EHR	Healthy	Neuro/Angio	Total
Healthy	537	1	538
Neuro/Angio	1	75	76
Total	538	76	614

κ 0.98 (95%CI 0.964-1.00, $p<0.001$)
